# Mapping paddy rice planting area in wheat-rice double-cropped areas through integration of Landsat-8 OLI, MODIS, and PALSAR images

**DOI:** 10.1038/srep10088

**Published:** 2015-05-12

**Authors:** Jie Wang, Xiangming Xiao, Yuanwei Qin, Jinwei Dong, Geli Zhang, Weili Kou, Cui Jin, Yuting Zhou, Yao Zhang

**Affiliations:** 1 Department of Microbiology and Plant Biology, and Center for Spatial Analysis, University of Oklahoma, Norman, OK, 73019, USA; 2 Institute of Biodiversity Science, Fudan University, Shanghai, 200433, China; 3 School of Computer Science and Information, Southwest Forestry University, Kunming, 650224, China

## Abstract

As farmland systems vary over space and time (season and year), accurate and updated maps of paddy rice are needed for studies of food security and environmental problems. We selected a wheat-rice double-cropped area from fragmented landscapes along the rural–urban complex (Jiangsu Province, China) and explored the potential utility of integrating time series optical images (Landsat-8, MODIS) and radar images (PALSAR) in mapping paddy rice planting areas. We first identified several main types of non-cropland land cover and then identified paddy rice fields by selecting pixels that were inundated only during paddy rice flooding periods. These key temporal windows were determined based on MODIS Land Surface Temperature and vegetation indices. The resultant paddy rice map was evaluated using regions of interest (ROIs) drawn from multiple high-resolution images, Google Earth, and in-situ cropland photos. The estimated overall accuracy and Kappa coefficient were 89.8% and 0.79, respectively. In comparison with the National Land Cover Data (China) from 2010, the resultant map better detected changes in the paddy rice fields and revealed more details about their distribution. These results demonstrate the efficacy of using images from multiple sources to generate paddy rice maps for two-crop rotation systems.

Studies on paddy rice fields aim to provide direct or indirect information for researches on food security, water resource management, and environmental sustainability. Paddy rice fields provide one of the main staple foods for more than half of the world’s population with 11% of cultivated land^1^. In Asia, the majority of rice agriculture relies on irrigation, accounting for 70% of global fresh water withdrawals^2^. Determining the area of paddy rice fields is an important component of obtaining more accurate information about agricultural water use to effectively manage fresh water resources^3^. In addition, as a kind of cultivated wetland, seasonally flooded paddy fields contribute 10–13% of the atmosphere’s anthropogenic methane^4^. Meanwhile, paddy rice fields are changing at a breakneck pace due to dramatic encroachment by expanding cities^5^ and the potentially reduced availability of water resources caused by climate change^6^. Therefore, it is urgently necessary to update and refine information about paddy rice planting areas in order to efficiently and accurately estimate crop production^7^, manage water resources^8^,^9^ and monitor greenhouse gas emissions^10^.

At the global and regional scales, several early studies of ecosystems and land cover have involved mapping paddy rice fields based on agricultural census data. In the late 1980s and early 1990s, several paddy rice datasets with coarse spatial resolution were produced to analyze global climate and greenhouse gas emission^11^,^12^. In the years following, two global cropland datasets representing crop patterns in the early 1990s and in the year 2000 were created at a spatial resolution of 5 arc minutes (~10 km)^13^,^14^. At the regional scale, a rice dataset for Asia was developed at the beginning of the 1980s^15^. In recent years, several studies on paddy rice planting areas were conducted by combining agricultural census data at the national scale^16^,^17^. Although most of the crop datasets were produced with input from multiple sources, these datasets were developed mainly by relying on statistics with coarse spatial resolution that were supplied by administrative units. Given the limitations of this spatial and temporal information, it is a challenge to apply these datasets to finer spatial research and to update them year to year.

Remote sensing is an efficient technique to acquire temporal and spatial cropland information repeatedly and consistently^18^. Historically, two main kinds of satellites have been used to map paddy rice fields: microwave and optical. Microwave satellites can penetrate through clouds and are thus superior for mapping paddy rice in regions dominated by long-term cloudy and rainy weather^19^,^20^,^21^,^22^, but available synthetic aperture radar (SAR) imagery is limited^19^ or expensive^23^. Commonly used optical sensors are the Multispectral Scanner System/Thematic Mapper/Enhanced Thematic Mapper Plus (MSS/TM/ETM+), the Moderate Resolution Imaging Spectroradiometer (MODIS)^24^,^25^,^26^,^27^,^28^,^29^, NOAA’s Advanced Very High Resolution Radiometer (AVHRR)^30^,^31^, and SPOT High Resolution Geometrical/High Resolution Visible Infrared/VEGETATION (HRG/HRVIR/VGT)^32^,^33^,^34^. Owing to continuous archiving and free to acquire, MODIS and Landsat have been the prevalent data sources of mapping paddy rice fields over the last several years.

Optical image classification techniques often use either individual image(s) or individual pixel(s) of time series data as input, here namely *image-based methods* and *pixel-based methods*. Image-based methods quantify the relationships (such as similarities or differences in spectra or texture) among all the pixels in an image for classification or object detection. These image-based methods have been applied to paddy rice mapping using single or multi-temporal optical images (e.g., MODIS and Landsat) at regional scales^23^,^35^,^36^,^37^. For image-based methods, collection of training samples (pixels) from ground reference data in each corresponding year remains a challenge^38^. Pixel-based methods rely primarily on the time series data for a pixel. These methods track the seasonal dynamics of a type of land cover and provide phenology information about the land surface. Several pixel-based algorithms have been developed to classify cropland using various optical images (e.g., MODIS, Landsat, SPOT, and FORMOSAT-2)^32^,^38^,^39^,^40^. To take into account the phenology of paddy rice, a pixel- and phenology-based algorithm using time series data of vegetation indices has been proposed. This was successfully applied to VGT and MODIS data for southern China and South and Southeast Asia^28^,^29^. Due to the high temporal resolution and continuous observation, MODIS data have frequently been combined with pixel- and phenology-based algorithms to track paddy rice phenology in order to map paddy rice areas or detect the intensity of paddy rice fields^24^,^26^,^27^,^41^,^42^,^43^. However, MODIS-based phenology information cannot capture the sub-pixel dynamics of small paddy rice fields in heterogeneous and fragmented agricultural landscapes^26^,^44^. This could be improved by using Landsat images with 30-m spatial resolution. Nevertheless, more research is needed to document the combination of Landsat images with pixel- and phenology-based algorithms for paddy rice mapping, especially in the case of Landsat 8 OLI images.

Reduced Landsat data availability caused by cloud cover or other problems may result in the failure of Landsat time series with 16-day intervals to distinguish the crops and trees. This problem is obvious in regions with double or multiple rotation agricultural systems, where cropland tends to be covered by plants year round, resulting in unavoidable confusion with natural evergreen forest. Fortunately, this problem can be solved by the Phased Array Type L-band Synthetic Aperture Radar (PALSAR) onboard the Advanced Land Observing Satellite (ALOS), given its capacity for forest detection^45^,^46^. The current study, which involves mapping paddy rice planting areas at a 30-m spatial resolution, has two aims: (1) to develop a pixel- and phenology-based algorithm by integrating time series optical images (Landsat-8, MODIS) and radar images (PALSAR) to map paddy rice planting areas in wheat-rice agricultural systems; and (2) to evaluate the potential utility of Landsat-8 OLI data in identifying fragmented paddy rice fields in complex agricultural landscapes. The case study area is located at the Yangzi-Huaihe Plain, China, which is characterized by a two-crop rotation (wheat and rice) agricultural system and intermixture of rural and urban landscapes (Figure S1).

## Results

### Spectral signatures of flooded pixels and other land cover types

**Figure 1:**
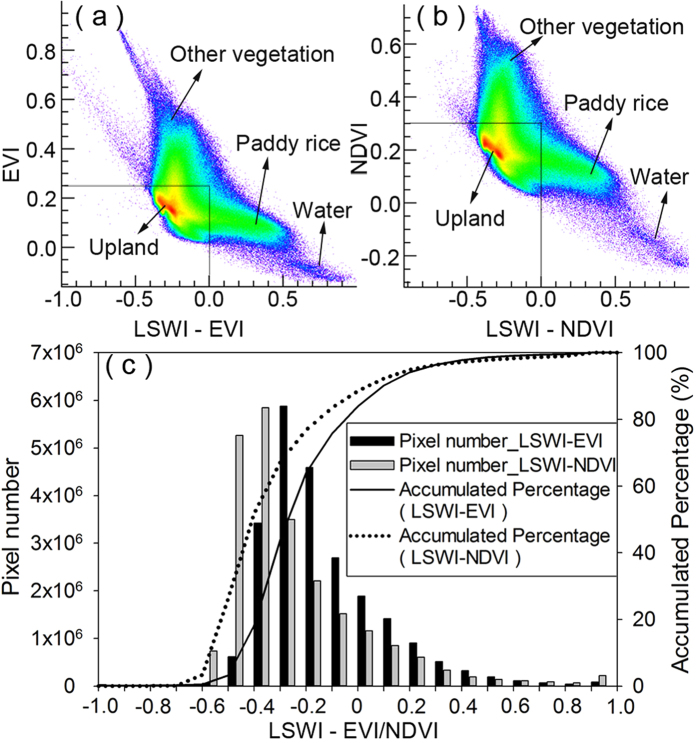
(**a**,**b**) show the gathering of four main objects (paddy rice fields, uplands, other vegetation, and water) in two-dimensional scatter plots: EVI and LSWI-EVI, and NDVI and LSWI-NDVI on Julian day 191(July 10, 2013). (**c**) Frequency histograms of LSWI-EVI and LSWI-NDVI. It shows that LSWI-EVI ≥ 0 detects more paddy rice fields than LSWI-NDVI ≥ 0. (**a**,**b**) created in ENVI 5.0, (**c**) created in Sigmaplot 12.0.

Different characteristics of vegetation indices are the basis for distinguishing flooded paddies from other land cover types. Figure S2 shows one example of the maps of Enhanced Vegetation Index (EVI), Normalized Difference Vegetation Index (NDVI), Land Surface Water Index (LSWI), LSWI-EVI, and LSWI-NDVI on Julian day 191 (July 10, 2013). At this time, paddy rice fields are in the midst of the flooding periods (including flooding/transplanting period and reviving period of paddy rice calendar), covered by a mixture of water and plants. Some uplands (e.g. corn fields) are going through the seeding or three leaves periods, covered by soil or by a mixture of soil and plants. The scatterplot graphs (Fig. 1(a,b)) show that EVI and NDVI mainly cluster between 0 and 0.3 for both the paddies and the uplands. The tremendous discrepancy between the two is that paddies cluster with LSWI-EVI ≥ 0 or LSWI-NDVI ≥ 0 and uplands cluster with LSWI-EVI < 0 or LSWI-NDVI < 0. Water bodies have LSWI-EVI ≥ 0 or LSWI-NDVI ≥ 0, but their EVI and NDVI are close to or less than zero. Other vegetation like forests or shrubs have higher EVI or NDVI (larger than 0.4) and lower LSWI-EVI or LSWI-NDVI (less than 0). Figure 1(c) shows that more paddy fields are detected by using LSWI-EVI ≥ 0 than LSWI-NDVI ≥ 0. In this image, about 10% of the pixels are flooded.

### Spatio-temporal dynamics of paddy rice fields

**Figure 2:**
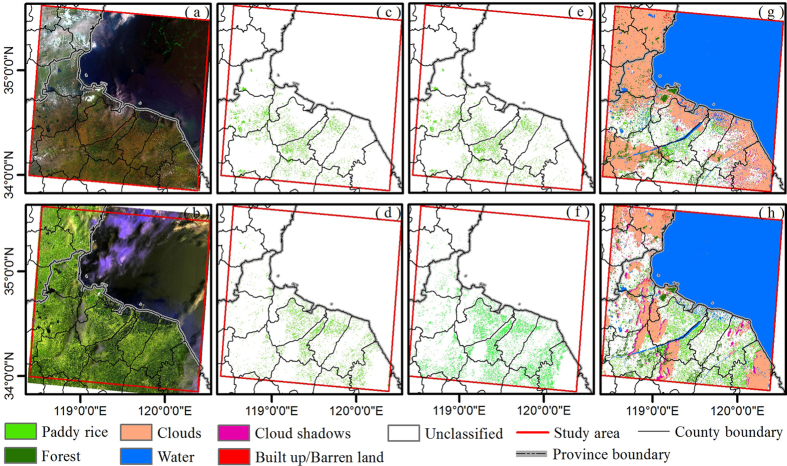
(**a**,**b**) Landsat-8 images after atmospheric correction on June 24, 2013, and July 10, 2013 (R,G,B = SWIR, NIR, Green); (**c**,**d**) Flooding pixels identified by the criteria LSWI-NDVI ≥ 0; (**e**,**f**) Flooding pixels identified by the criteria LSWI – EVI ≥ 0; (**g**,**h**) Paddy rice mappings identified by the criteria LSWI-NDVI ≥ 0 or LSWI - EVI ≥ 0. Clouds/cloud shadows, water, built-up/barren land, forest are all shown. Map created in ArcMap 10.1. (**a**,**b**) were downloaded from Earth Resources Observation and Science (EROS) Center, USGS ( http://earthexplorer.usgs.gov/).

Two Landsat-8 images (June 24 & July 10, 2013) were acquired during the flooding periods. Figure 2(a,b) show the original images with bands combination: R: SWIR1, G: NIR, B: Green. Figure 2(c,d,e,f) represent relevant paddy rice maps identified by the criteria LSWI-NDVI ≥ 0 and by LSWI-EVI ≥ 0, respectively. Figure 2(g,h) are the combined paddy rice maps identified by the criteria LSWI-NDVI ≥ 0 or LSWI-EVI ≥ 0. They also show cloud/cloud shadow, water body, built-up/barren land, and forest masks. Because of less cloud/cloud shadow cover, the image quality on July 10 is better than the one on June 24 and more paddy rice fields are detected from it.

### Spatial distribution of paddy rice fields

**Figure 3:**
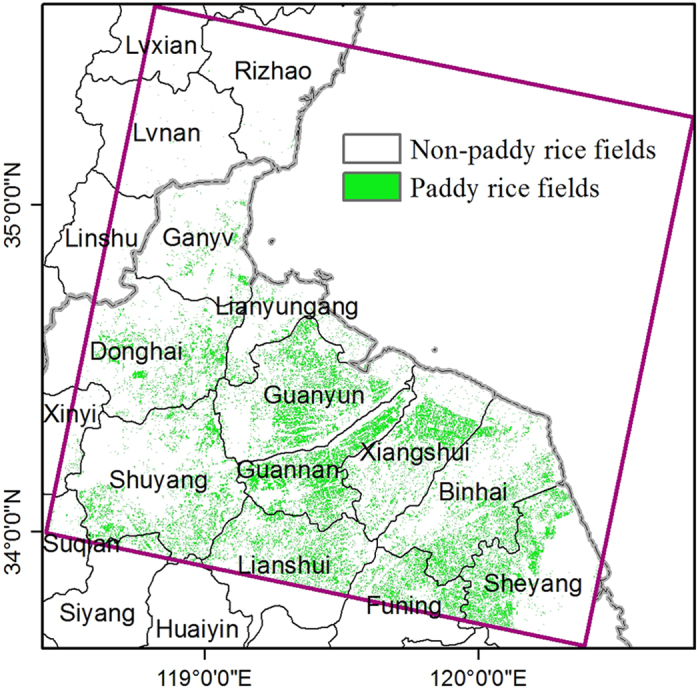
The paddy rice planting area map at 30 m spatial resolution, identified through the criteria LSWI - NDVI ≥ 0 or LSWI - EVI ≥ 0 from Landsat-8 images on June 24, 2013, and July 10, 2013. The total number of paddy rice plots is 180,135, and the mean paddy rice field size is 0.0238 km^2^ (~154 m^*^154 m). Map created in ArcMap 10.1.

Figure 3 shows the spatial distribution of paddy rice fields in 2013 at 30 m spatial resolution, which integrates the paddy rice maps from June 24 and July 10. Few paddy rice fields are detected within the Shandong Province. However, in the Jiangsu Province, paddy rice fields are distributed widely and extensively. The paddy rice planting area is estimated to be approximately 2406.0 km^2^, accounting for 37.6% of the study area. Additionally, this map reveals that the rice agriculture here is mainly conducted in small or medium size croplands. We used the Fragstats software 4.2 ( http://www.umass.edu/landeco/research/fragstats/downloads/fragstats_downloads.html#diagnostic) to evaluate the fragmentation of the paddy rice fields. In this map, the total number of paddy field patches is 180,135, and the mean patch size of paddy fields is 0.0238 km^2^ (~154 m × 154 m).

### Evaluation of Landsat-derived rice map

In this work, 3,610 paddy rice pixels (199 ROIs) and 3,113 non-paddy rice pixels (85 ROIs), located in good observation regions, were employed to calculate the confusion matrix (Table 1).

The validation showed the paddy rice/non-paddy rice map has a reasonably high accuracy. The paddy rice planting area in this map yielded 83.1% producer accuracy and 97.5% user accuracy. The non-paddy rice area in this map has 97.6% producer accuracy and 83.3% user accuracy. The overall accuracy and Kappa coefficient of this map are 89.8% and 0.79, respectively.

### Comparison to other available datasets

**Figure 4:**
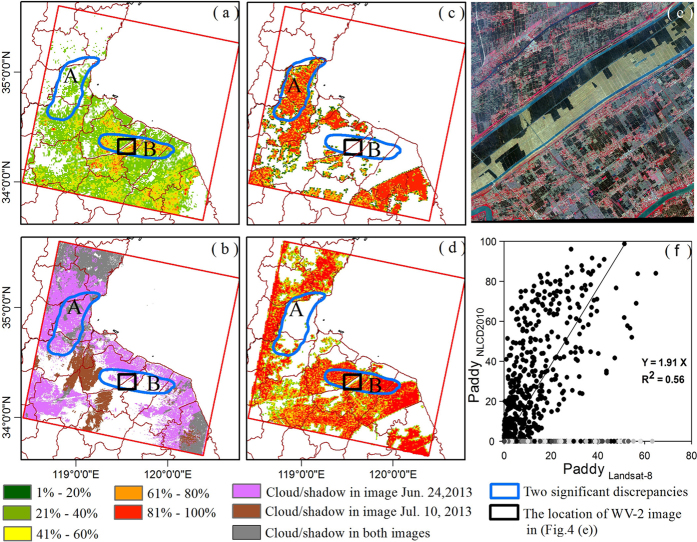
(**a**) 1 km Landsat-8 paddy rice map (Paddy_Landsat-8_), and different colors presenting the occupation levels of paddy rice fields in given pixels; (**b**) The cloud/cloud shadow masks for images on June 24 and July 10, 2013; (**c**) Paddy rice map of 1 km NLCD2010 dataset (Paddy_NLCD2010_); two significant discrepancies between Paddy_Landsat-8_ and Paddy_NLCD2010_ are marked with blue circles (A, B); (**d**) Upland map of 1 km NLCD2010 dataset; (**e**) WorldView-2 image from June 20, 2012, the location of which is marked by the black rectangle in (**a**, **b**, **c**, **d**). It shows abundant flooded signals in the croplands, which should be classified as paddy rice fields just as in the results of this study, rather than uplands in NLCD2010. (**f**) Pixel-level comparison between these two datasets. The solid line was drawn from the regression analysis of all the points except the grey ones gathering on the X-axis. These grey points reveal that Paddy_Landsat-8_ detected new paddy rice fields that were absent in Paddy_NLCD2010_. Maps (**a**, **b**, **c**, **d**, **e**) created in ArcMap 10.1 and Map (**f**) created in SigmaPlot 12.0. Image (**e**) was provided by NASA for use in the NASA projects.

To compare this result with NLCD2010 (1 km gridded 2010 National Land Cover Data, China) Paddy data, the 30 m Landsat-8 paddy rice planting area mapping was resampled to have 1 km spatial resolution (Paddy_Landsat-8_, Fig. 4(a)). In general, the spatial aggregation of paddy rice fields in both mappings (Fig. 4(a,c)) was consistent except for two areas marked with blue circles (A, B). Furthermore, Paddy_Landsat-8_ revealed more details about paddy rice field patterns than Paddy_NLCD2010_ data (Fig. 4(c)).

The total area of paddy rice planting in 2013 estimated by Paddy_Landsat-8_ was 2406.0 km^2^. It was far less than the paddy rice area (4,986.96 km^2^) in 2010 estimated by Paddy_NLCD2010_. Just considering the paddy rice fields in good observation regions, the paddy rice area from Paddy_Landsat-8_ was 1,311.14 km^2^, 12.2% lower than that (1,492.85 km^2^) from Paddy_NLCD2010_. Figure 4(b) shows the cloud/cloud shadow masks of images on June 24 and July 23, 2013. For the difference marked by the blue circle A, one obvious explanation is cloud contamination on June 24, 2013 (Fig. 4(b)). Likewise, visual analysis showed a large difference occurred in the blue circle B (Fig. 4(a,c)). Paddy_Landsat-8_ detected some paddy rice fields in this area, while NLCD2010 data classified these croplands as uplands (Fig. 4(d)). One WordView-2 image (Fig. 4(e)) on June 20, 2012, its location marked by the black rectangle, was used to judge the performance of these two results. It shows there are abundant flooding signals in the croplands that should be classified as paddies rather than uplands.

At the pixel level, the regression analysis was carried out to compare these two datasets for the paddy rice area estimate. R-square was 0.56 without considering the grey points gathering on X-axis (Fig. 4(f)). One possible reason for this difference was that Paddy_Landsat-8_ detected new paddy rice fields that were absent in Paddy_NLCD2010_, just as the paddy rice fields within the blue circle B. In addition, owing to Paddy_NLCD2010_ referring to the paddy rice planting area in 2010 and Paddy_Landsat-8_ focusing on that in 2013, there were uncertainties due to paddy rice changes in actual agricultural practice or rapid Land Use/Land cover changes.

## Discussion

**Figure 5:**
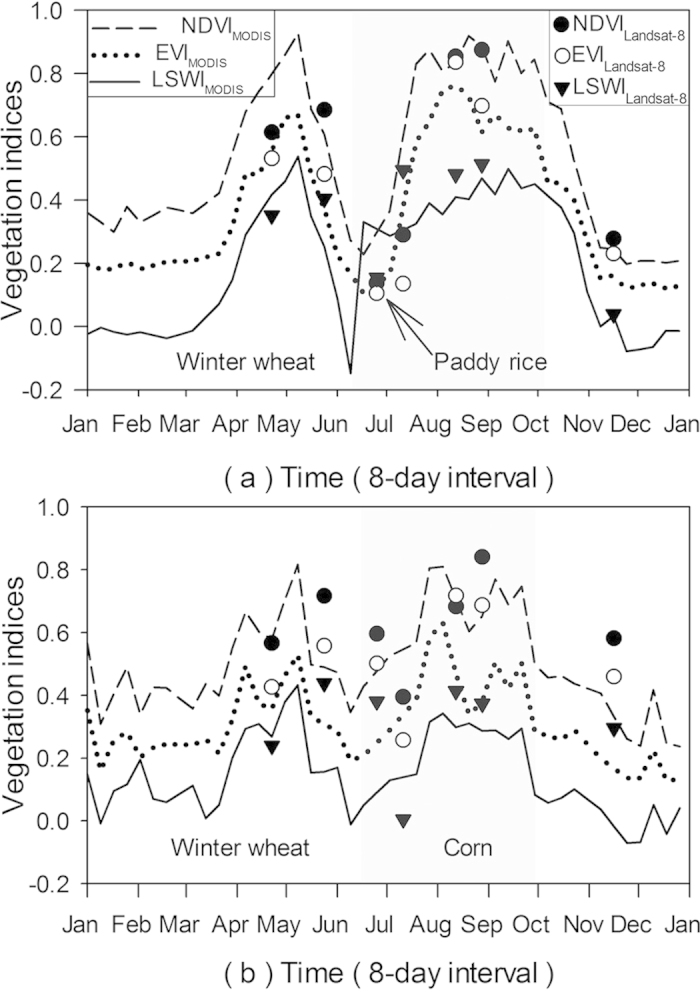
The seasonal dynamics of NDVI, EVI, and LSWI, extracted from MOD09A1 product in 2012 and Landsat-8 images with good quality observations in 2013, for two-crop rotation agricultural systems. (**a**) Winter wheat and paddy rice rotation (34.286 °N, 119.642 °E), (**b**) winter wheat and corn rotation (34.237 °N, 119.232 °E).

In this study, multi-source remote sensing data including Landsat-8, MODIS, and PALSAR were used to identify paddy rice fields from the rotation agricultural system of winter wheat and paddy rice. 30 m Landsat OLI images provide more details about the distribution of paddy rice fields (Fig. 4(a)), and they are also helpful in identifying paddy rice from heterogeneous crops, which are common in this study area (Figure S5). MODIS-based LST_night_ images have the ability to track the growing season, which is consistent with the results of climate observations (Figure S3). The dynamics of MODIS-based vegetation indices give it the ability to detect the flooding signals (including flooding/transplanting and reviving periods) of paddy rice fields (Fig. 5(a)) and yield results consistent with the crop calendar (Figure S4). Therefore, MODIS-based LST_night_ and vegetation indices (VIs) dynamics can help automatically select Landsat-8 images for paddy rice planting area mapping. PALSAR data have advantages in mapping forest, which can be used to generate forest masks in paddy rice mapping to solve the mixture of croplands and forest, especially in the areas with long growing seasons. Therefore, the combination of multi-source remote sensing data makes it possible to map paddy rice fields automatically in complicated agricultural systems.

The integration of Landsat-8, MODIS, and PALSAR data also showed some advantages in the identification of fragmented paddy rice fields. The mean patch size of paddy fields in the study area was 154 m × 154 m in 2013. In terms of relatively small and even fragmented rice fields, the Landsat-8 images with finer spatial resolution (30 m) were better than the MODIS satellite data (500 m) in reducing mixed-pixel problems. In last several decades, the croplands in this study area were becoming fragmented rapidly, because of high population pressure, rapid economic development, urbanization, and limited arable lands. In 1983, the mean farmland area was about 14 km^2^, reduced to 6 km^2^ in 2000^47^. In neighboring districts, the mean patch size of paddy rice fields also showed rapid diminution, declining from ~12 km^2^ in 1990 to ~1.4 km^2^ by 2006^48^. At the same time, the minimum patch area was also reduced from 2 × 10^−4^ km^2^ in 1990 to 5 × 10^−5^ km^2^ in 2006. Cropland fragmentation is common in Asia^40^. In China, the crop land area per household was 5.3 × 10^−3^ km^2^ divided into 6.06 plots on average^49^. The net cultivated area per capita was lower than 6 × 10^−4^ km^2^ in Bangladesh^50^, and the average rice field was 1.1 × 10^−2^ km^2^ in Taiwan^32^. Therefore, the integration of multi-source remote sensing data has the potential to provide more valuable information for updating and refining paddy rice maps in Asia.

We recognized that the paddy rice map identified in this study was affected by several potential factors. The first source of uncertainty was the limited availability of images from the 16-day revisit Landsat-8 satellite that detected the flooded signals of paddy rice fields. In the study area, the flooding periods usually lasted around three weeks, from mid to late June or early July. Therefore, the flooded signals of paddy rice fields could only be observed by one or two images. This shortage of image data could be remedied in the future by using multi-year Landsat-8 data and other optical sensors, such as Landsat TM/ETM+, and Sentinel-2A/B^51^,^52^. Secondly, paddy rice planting area mapping based on Landsat-8 images did not avoid the impacts of clouds and their shadows, just as the other optical sensors, MODIS, TM, AVHRR^27^,^30^,^53^. Figure 2(a,b) show that the original images from June 24 and July 10 are covered with about 30% cloud. In this study, Fmask was used to detect clouds and cloud shadows. Fig. 2(g,h) show that Fmask worked well, but it overestimated the clouds. Therefore, these factors would result in the underestimation of paddy rice fields. Although 30 m Landsat-8 images were used in this study, it was still a challenge to remove the influence of the mixed pixels (e.g. vegetation and water). These pixels had the same characteristics as paddy rice fields during the flooding periods. Therefore, they might be identified as paddies if the ratio of vegetation and water satisfied the extraction algorithm: LSWI – EVI ≥ 0 or LSWI –NDVI ≥ 0. The confusion of paddy fields with other land cover types caused by mixed pixels is a common problem occurring in various sensors, including Landsat TM/ETM, SPOT, MODIS^32^,^33^. Various agricultural practices would be another source of uncertainty. This research extracted flooded paddy rice fields with Landsat-8 images from June 24 and July 10, 2013. If some farmers deviated from the regular agricultural practice calendar, their paddy rice fields with earlier or later plants would not be detected from these two images, because flooding/transplanting signals would be weak or nonexistent. In addition, the rapid Land Use/Land Cover changes caused by urbanization and industrialization in this area from 2010 to 2013 was one of the reasons for the discrepancy between Paddy_Landsat-8_ and Paddy_NLCD2010_.

The results of this study have demonstrated the potential of multi-source remote sensing data (Landsat-8, MODIS and PALSAR) to map paddy rice planting areas in the wheat-rice double cropping system, using a pixel- and phenology-based algorithm. MODIS-based LST_night_ and VIs dynamics make it possible to automatically select Landsat-8 images within key time windows. PALSAR data can solve the mixture of croplands and forest. Landsat-8 images provide more details about the distribution of paddy rice fields, which is useful for the extraction of fragmented ones. As Landsat-8, MODIS and PALSAR Forest/Non-forest product are available to the public, there is a potential to develop 30 m paddy rice planting area maps across the two-crop zone using this approach.

## Methods

### Maps of non-cropland land cover types

**Figure 6:**
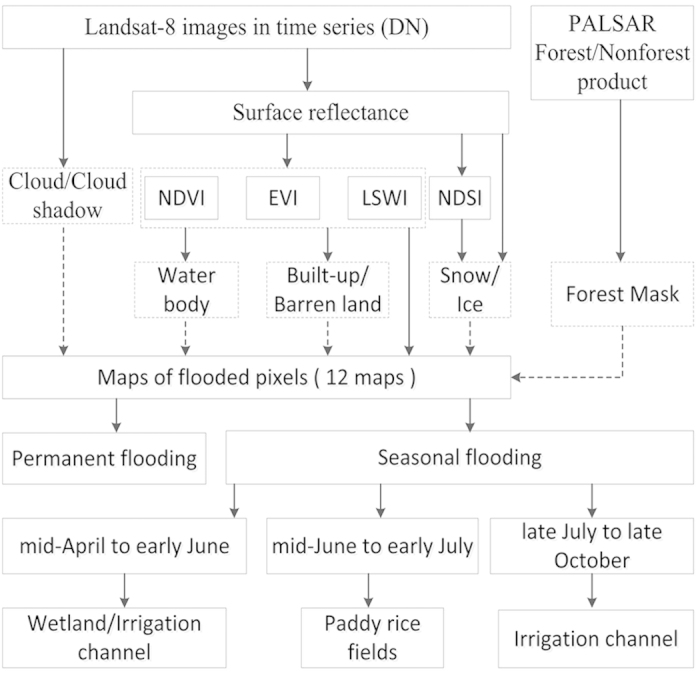
The workflow for mapping paddy rice planting area in a doubling agricultural system (Yangzi-Huaihe Plain) using Landsat-8 images from 2013. PALSAR 50 m FNF product was used as forest mask. Vegetation indices algorithms were used to detect non-croplands and flooded croplands. Figure created in Microsoft Visio 2010.

It is necessary to map some major non-cropland land covers, including water bodies, built-up and barren lands, forests, permanently flooded regions during the growing season (Fig. 6). In practice, these land cover types potentially affect the seasonal dynamics of vegetation indices and the accuracy of the paddy rice detection algorithm.

Water bodies have lower NDVI and EVI and higher LSWI values. Similar to the water body extraction algorithm based on MODIS^29^, pixels in each image meeting the condition NDVI < 0.1 and NDVI < LSWI were extracted as water. Persistent water bodies were then composed of the pixels that were identified as water in all the good quality observations throughout the plant growing season.

Built-up and barren lands have high reflectance at visible and near infrared bands and low moisture content. Based on these physical features, a simple algorithm was put forward for built-up/barren lands, that is, LSWI < 0. Then, we calculated the frequency of a pixel identified as barren/built-up lands in the Landsat-8 time series (the total number of good quality observations). Permanent built-up/barren land mask was made up by pixels with a frequency of ≥90%.

Forest cover can be mapped from optical images or microwave images. Because of frequent cloud cover and 16-day revisit cycle, there is no sufficient number of Landsat images available in 2013 to distinguish croplands and forests. Previous studies showed that the cloud-free L-band SAR is the most advantageous for forest detection^46^. In this research project, the PALSAR-50 m Forest/Non-forest (FNF) classification map from 2010, provided by Japan Aerospace Exploration Agency (JAXA), were resampled to generate a 30 m forest mask. These PALSAR 50 m products are free to the public at the official ALOS Kyoto and Carbon Initiative website ( http://www.eorc.jaxa.jp/ALOS/en/palsar_fnf/fnf_index.htm)^54^. Compared to the ground truth data, these forest/non-forest products yielded 84% total accuracy on average^55^. This forest mask includes evergreen and deciduous forests.

Unlike seasonally flooded paddy rice fields, some lands remain flooded during the whole growing season. Therefore, it is necessary to distinguish between these two kinds of flooded lands. First, this work identified the flooded pixels for each Landsat-8 image following the rule LSWI – EVI ≥ 0 or LSWI – NDVI ≥ 0. Then, a map of the permanently flooded lands was produced from the pixels flooded in all the good-quality observations throughout the growing season.

According to the seasonal dynamics of MODIS-based vegetation indices (Fig. 5(a)) and Landsat-8 datasets (Table S1), seasonally flooded pixels were further divided into three phases: flooded from mid-April to early June, from mid-June to early July, and from late July to early November. From mid-April to early June, flooded signals mainly appeared in natural wetlands, some aquaculture areas, and irrigation channels. From mid-June to early July, paddy rice fields begin flooding and transplanting with significant flooded signals. From late July to early November, flooded signals disappear from paddy rice fields and they may appear in the irrigation channels of croplands.

### Algorithms for identifying inundation and paddy rice fields

Paddy rice is usually planted in flooded fields. Three periods can be differentiated during paddy rice growth. In the flooding periods, the land surface of paddy rice fields is covered by water with a depth of 2-15 cm and green paddy rice plants. About 50 to 60 days later, most of the cropland surface is covered by the canopies of paddy rice plants. At the end of the growth period prior to harvesting, there is a decrease in the number of leaves and a decrease of leaf and stem moisture content.

The phenological features of main crops in the study area were investigated through MOD09A1 8-day composite vegetation indices time series (Fig. 5), obtained from the MODIS data portal at the Earth Observation and Modeling Facility (EOMF), University of Oklahoma ( http://www.eomf.ou.edu/visualization/gmap/). To get reliable vegetation index time series data, the bad-quality observations were gap-filled through a three-step gap-filling procedure^56^. Figure 5(a,b) show that the largest difference between paddy rice and other crops is the flooded signal during the growing season.

The phenological features of main crops were also investigated via the dynamics of three vegetation indices based on Landsat-8 time series images. Figure 5(a) shows that LSWI is larger than EVI in early July. Figure 5(a,b) indicate that despite a 16-day revisit, it is possible to detect the difference between paddy rice and corn from the Landsat-8 images. According to the seasonal dynamics of NDVI, EVI, and LSWI and the finer spatial resolution of Landsat-8 images, an algorithm was proposed to extract paddy rice fields from 30 m Landsat-8 images: LSWI – NDVI ≥ 0 or LSWI – EVI ≥ 0. According to the dynamics of MODIS vegetation indices, Landsat-8 images acquired within the flooding periods were used to map paddy rice fields. In this study, the flooding periods of paddy rice fields was from mid-June to early July, composed by the flooding/transplanting period and the reviving period of paddy rice calendar.

### Accuracy assessment of resultant maps

We used ground truth data (field photos (Figure S5)), Google Earth (GE), and high-resolution images to locate and digitize ROIs. Google Earth displays high-resolution images, which have been used to validate land cover classification in several studies^57^,^58^,^59^. However, GE images were not enough to visually interpret ROIs as it lacked images within key time windows. We also ordered multiple high-resolution images from 2012 and 2013 from the NASA Goddard Space Flight Center, including WorldView-2 (WV2), OrbView5 (OV5), and QuickBird2 (QB2). According to the reference information, we generated a series of random sampling points and interpreted them into ROIs. In total, 15,751 Landsat-8 pixels were acquired, including 7,388 paddy rice pixels (173 ROIs) and 8,363 non-paddy rice pixels (427 ROIs) (Figure S6).

The accuracy of the paddy rice map produced in this study was assessed by using the “Ground Truth ROIs” method in ENVI software. We obtained a confusion matrix between the paddy rice map and the ROI data, and producer’s accuracy, user’s accuracy, overall accuracy, and the Kappa coefficient. We estimated the accuracy of a paddy rice map using only good quality observations during the flooding/transplanting period. Finally, according to the cloud/cloud shadow masks from June 24 and July 10, we selected 3,610 paddy rice pixels (199 ROIs) and 3,113 non-paddy rice pixels (85 ROIs) located in good observation regions that were then used to validate the final paddy rice map.

### Comparison with other available datasets of paddy rice fields

We compared our results with NLCD2010 to analyze their differences and the dynamics of paddy rice fields. The paddy rice map was compared with NLCD2010 at two scales. At the regional level, we analyzed the variations of paddy rice fields in spatial distribution and planting areas. At the pixel level, correlation analysis was used to compare these two datasets in regard to the estimation of paddy rice planting areas.

## Author Contributions

X.-M.X. and J. W. designed the research. J.W., Y.-W. Q. and Y.-T.Z. collected field data. J.W., J.-W.D, G.-L.Z., W.-L.K. and C. J. performed data analysis. Y. Z. contributed to the writing of the manuscript. All authors commented on the manuscript.

## Additional Information

**How to cite this article**: Wang, J. *et al.* Mapping paddy rice planting area in wheat-rice double-cropped areas through integration of Landsat-8 OLI, MODIS, and PALSAR images. *Sci. Rep.* **5**, 10088; doi: 10.1038/srep10088 (2015).

## Supplementary Material

Supplementary Information

## Figures and Tables

**Table 1:** Accuracy assessment of the 30 m Landsat-8 paddy rice map using ROIs in the Yangzi-Huaihe Plain, southeast China.

	**Ground Truth**			
	**Paddy rice**	**Non-paddy rice**	**Total**	**User Acc.**
paddy rice	3001	76	3077	97.5%
Non-Paddy rice	609	3037	3646	83.3%
Total	3610	3113	6723	
Pro. Acc	83.1%	97.6%		
Overall accuracy	89.8%	Kappa coefficient	0.79	
