# Supplementary material for: Mapping paddy rice planting area in wheat-rice double-cropped areas through integration of Landsat-8 OLI, MODIS, and PALSAR images
Source: Sci Rep. 2015 May 12;5:10088. doi: 10.1038/srep10088 (PMC4428029; doi:10.1038/srep10088)
Supplement: Supplementary Information [file srep10088-s1.doc]

**Supporting Information for**

**Mapping paddy rice planting area in wheat-rice double-cropped areas through integration of Landsat-8 OLI, MODIS, and PALSAR images**

Jie Wang^1 *^, Xiangming Xiao^1,2 *^ , Yuanwei Qin^1^, Jinwei Dong^1^, Geli Zhang^1^, Weili Kou^1,3^, Cui Jin^1^, Yuting Zhou^1^ and Yao Zhang^1^

^1^Department of Microbiology and Plant Biology, Center for Spatial Analysis, University of Oklahoma, Norman, OK, 73071, USA

^2^Institute of Biodiversity Science, Fudan University, Shanghai, 200433, China

^3^Department of Computer and Information Science, Southwest Forestry University, Kunming, Yunnan, 650224, China

*Corresponding author:

Prof. Xiangming Xiao

Department of Botany and Microbiology, College of Arts and Sciences

Associate Director, Center for Spatial Analysis, College of Atmospheric & Geographic Sciences

Email: [xiangming.xiao@ou.edu](mailto:xiangming.xiao@ou.edu); Telephone: (405)-3258941

Website: [http://www.eomf.ou.edu](http://www.eomf.ou.edu/)

Jie Wang

Department of Microbiology and Plant Biology

College of Arts and Sciences, University of Oklahoma

101 David L. Boren Blvd.

Norman, OK 73019, USA

Email: [jiewang@ou.edu](mailto:jiewang@ou.edu)

Materials and Methods

Study area

This study focused on a coastal area (33°06′14″ - 35°06′15″ N, 118°04′58″ - 120°03′28″ E), composed of the northeast Jiangsu province and the southeast Shandong province, a part of the Yangzi-Huaihe plain of China, at OLI image path 120 and row 36 (Figure S1). The area is flat with an average elevation of about 17 meters. It is comprised of 19 counties, three in the Shandong province and the rest in the Jiangsu province. Only seven counties, belonging to the Jiangsu province, are located completely within the research area.

Within a warm temperate and sub-humid monsoon climate zone^1^, there is a long crop growing season in the test area. Records of averages for three years (2010-2012) from four local National Weather Stations demonstrate that the 8-day mean air temperature is usually higher than 10 ˚C from late March to early November (Figure S3 (a)) and precipitation mainly occurs from late June to late September. The crop growing season is around DOY (Day of Year) 110 to 295, according to the criterion that the first and last date of the 8-day night Land Surface Temperature (LSTnight) is greater than 5˚C (Figure S3 (b), (c)), calculated from the MYD11A2 product in 2010. Limited by temperature and precipitation, there is one rice crop per year in this area. However, two-crop rotation systems are dominant. In the southern part of the test area within the Jiangsu province, the double cropping system mainly consists of a winter wheat and paddy rice rotation, a rapeseed and paddy rice crop rotation^2^, or a winter wheat and corn rotation. In the northern test area within the Shandong province, the double cropping system is mainly a winter wheat and corn rotation.

According to three years-worth (2010-2012) of observation records from two agricultural meteorology and phenology stations (Ganyv and Lvxian), a local crop calendar was made for three main crops: winter wheat, paddy rice, and corn (Figure S4). Varying both inter-annually and inter-regionally, winter wheat generally matures in early or mid-June, and the fields are flooded within 1-2 weeks after a quick harvest of winter wheat. One week to 10 days later, usually in mid-to-late June, rice seedlings are transplanted in this flooded soil. The harvest of rice usually happens in late October and then the next crop rotation begins. After the harvest of winter wheat, corn, as another dominant crop, is usually sown in late June and harvested in late September. Therefore, from June to October, paddy rice, corn, and other crops (peanut, soybean) constitute mixed agricultural landscapes. The surface water bodies in this region include mainly lakes, rivers, and salt ponds, as well as fish and aquaculture ponds.

Landsat-8 image data

# The Landsat-8 satellite, launched on February 11, 2013, by NASA, carries two sensors, the Operational Land Imager (OLI) and the Thermal Infrared Sensor (TIRS). The OLI collects images with six narrower heritage bands and two newly specified bands: a deep blue band (Band 1, 0.43-0.45µm) observing ocean color in coastal zones and a shortwave infrared band (band 9, 1.36-1.39µm) detecting cirrus clouds. Images are collected at 16-day intervals with 15-meter panchromatic and 30-meter multi-spectral spatial resolutions. TIRS measures land surface emitted radiance in two thermal infrared bands with a spatial resolution of 100 m. These two sensors provide images with 12-bit radiometric resolution and higher signal-to-noise.

# In this study, 12 standard level 1T data products (path/row 120/36), acquired from April 21, 2013, to December 01, 2013, were obtained from USGS by EarthExplorer (<http://earthexplorer.usgs.gov/>) (Table S1). The Landsat-8 level 1 GeoTIFF data products have finished radiometric calibration, systematic geometric correction, precision correction, and parallax error correction^3^.

Atmospheric correction

# Atmospheric correction was carried out for each image by using the updated Landsat Ecosystem Disturbance Adaptive Processing System (LEDAPS) routine. The LEDAPS project ingests and calibrates digital numbers to at-sensor radiance and then to surface reflectance after atmospheric correction by using the 6S approach^4^. This radiative transfer code has an accuracy better than 1% over a range of atmospheric stressing conditions^5^.

Cloud and cloud shadow identification

# Clouds and associated cloud shadows obstruct the view of the land surface, frequently bringing false information into the land cover analyses^6,7^. To remove these interference factors, Fmask (Function of mask) code was used to generate cloud and cloud shadow masks for each of the Landsat-8 images. Fmask is one of the detection methods based on clouds’ physical properties at a global scale. Cloud shadow layers are generated by a sequential processing, including darkening effect identification of the cloud shadow in the NIR band, segmentation of potential cloud layer, and geometric match of the potential cloud shadow. The average detection accuracy for clouds and for cloud shadows is more than 96% and 70%, respectively^8^.

# In this paper, a good quality observation means that the pixels in each image are not covered by clouds or cloud shadows. Using the cloud and cloud shadow masks, the quality of each image and its percentage of good observations were obtained and calculated (Table S1). From April to December, there were eight valid, informative images with a percentage of good observations larger than 50%.

Vegetation indices

# Four vegetation indices were calculated from the surface reflectance. As a vegetation measure, Normalized Difference Vegetation Index (NDVI)^9^ successfully monitors seasonal and inter-annual changes in vegetation growth and activity^10^. Enhanced Vegetation Index (EVI) was developed to improve vegetation monitoring in high biomass regions^10,11^. Both can provide green-related vegetation information. Land Surface Water Index (LSWI), developed by SWRI and NIR spectral bands, is a water-sensitive vegetation index, tracking water thickness in vegetation and soil^12,13^. Normalized Difference Snow Index (NDSI) was developed to detect snow automatically^14,15^.

$NDVI=\frac{\rho_{NIR}-\rho_{red}}{\rho_{NIR}+\rho_{red}}$ (1)

$EVI=2.5\times\frac{\rho_{NIR}-\rho_{red}}{\rho_{NIR}+6\times\rho_{red}-7.5\rho_{blue}+1}$ (2)

$LSWI=\frac{\rho_{NIR}-\rho_{SWIR}}{\rho_{NIR}+\rho_{SWIR}}$ (3)

$NDSI=\frac{\rho_{green}-\rho_{SWIR}}{\rho_{green}+\rho_{SWIR}}$ (4)

Where $\rho_{blue}$, $\rho_{green}$, $\rho_{red}$, $\rho_{NIR}$ and $\rho_{SWIR}$ are the surface reflectance values of blue, green, red, near-infrared, and shortwave-infrared bands. Differences exist among various sensors on band sets, for Landsat-8: Blue (450-515nm), Red (630-680nm), NIR (845-885nm), and SWIR (1560-1660nm).

Snow and ice covers

# Owing to high surface reflectance in the visible spectral bands, snow cover could potentially impact the seasonal dynamics of vegetation indices, especially in winter and spring^16^. In this research, snow cover masks were generated based on the MODIS snow product algorithm^14,15^. Snow-covered pixels were identified through NDSI > 0.4 and NIR > 0.11 from each Landsat-8 image.

Field survey data

# To estimate the classification accuracy of this work, a field survey was conducted in the summer from July 21, 2013, to August 7, 2013. In general, the croplands were fragmented and agricultural landscapes were heterogeneous. In this survey, field photos were taken at 16 sites with GPS cameras (Figure S5), including 6 paddy rice fields, 2 cornfields, 1 mixture cropland of paddy rice and corn, and 7 other croplands (e.g. peanut fields). The sites with large croplands were chosen for taking photos. At each sample site, at least five photos were taken from within the croplands, one each towards the four cardinal directions (north, east, south and west) and a vertical-down view. All the photos taken over that summer were submitted to the field photo library of the Earth Observation and Modeling Facility at the University of Oklahoma (<http://www.eomf.ou.edu/photos/browse/>) as public property; they can be used by anyone who is interested. During this field trip, we talked to local farmers to gather additional information on the cropping calendar, crop rotation, and management practices such as fertilization and irrigation. From the field photo library, we also selected field photos from an additional 20 sites that were taken by other people.

High-resolution images

# Google Earth images were not enough to visually interpret ROIs as it lacked images within key time windows. We also ordered multiple high-resolution images from 2012 and 2013 from the NASA Goddard Space Flight Center, including WorldView-2 (WV2), OrbView5 (OV5), and QuickBird2 (QB2). The WV2 satellite sensor provides a 0.5m panchromatic band and eight multispectral bands with 1.8m resolution at Nadir (<http://www.satimagingcorp.com/satellite-sensors/worldview-2.html>). The OV5 collects a 0.41m panchromatic and four 1.65 m multispectral images at Nadir (http://www.satimagingcorp.com-/satellite-sensors/geoeye-1.html). The QB2 provides a 0.61m panchromatic and four 2.4m multispectral images at Nadir (<http://www.satimagingcorp.com/satellite-sensors/quickbird.html>). Figure S6 showed the distribution of high-resolution images used in this study as well as acquisition time and satellite sensors. Then, according to the reference information, we generated a series of random sampling points and interpreted them into ROIs. In total, 15,751 Landsat-8 pixels were acquired, including 7,388 paddy rice pixels (173 ROIs) and 8,363 non-paddy rice pixels (427 ROIs) (Figure S6).

2010 National Land Cover Data

The NLCD was developed by the Chinese Academy of Sciences using remote sensing and geographic information system techniques^17,18^. The 2010 1:100,000 NLCD was interpreted from 2010 Landsat TM digital images by using the human-computer interactive interpretation method. For the areas that do not have TM data, the China–Brazil Earth Resources Satellite (CBERS) and the Huanjing-1 satellite (HJ-1) were used as supplemental data to fill gaps. To ensure high-quality and consistent NLCD, nationwide field surveys and uniform quality control were conducted before and after the development of each dataset. A classification system of 25 land cover classes was applied in the NLCD project, including paddy land and upland. The visual interpretation vector dataset was converted into a 1-km gridded database. Each 1-km gridded cell had the percentage of the area of various land-use types within it.

1. Fangquan, M. *et al.* Rice Cropping Regionalization in China. *Chinese J. Rice Sci.* **2**, 97-110 (1988).

2. Xiao, X. *et al.* Landscape-scale characterization of cropland in China using Vegetation and landsat TM images. *Int. J. Remote Sens.* **23**, 3579-3594 (2002).

3. Roy, D. P. *et al.* Landsat-8: Science and product vision for terrestrial global change research. *Remote Sens. Environ.* **145**, 154-172 (2014).

4. Masek, J. G. *et al.* A Landsat surface reflectance dataset for North America, 1990-2000. *Ieee Geosci. Remote Sens. Lett.* **3**, 68-72 (2006).

5. Ju, J. C., Roy, D. P., Vermote, E., Masek, J. & Kovalskyy, V. Continental-scale validation of MODIS-based and LEDAPS Landsat ETM plus atmospheric correction methods. *Remote Sens. Environ.* **122**, 175-184 (2012).

6. Goodwin, N. R., Collett, L. J., Denham, R. J., Flood, N. & Tindall, D. Cloud and cloud shadow screening across Queensland, Australia: An automated method for Landsat TM/ETM plus time series. *Remote Sens. Environ.* **134**, 50-65 (2013).

7. Huang, C. Q. *et al.* Automated masking of cloud and cloud shadow for forest change analysis using Landsat images. *Int. J. Remote Sens.* **31**, 5449-5464 (2010).

8. Zhu, Z. & Woodcock, C. E. Object-based cloud and cloud shadow detection in Landsat imagery. *Remote Sens. Environ.* **118**, 83-94 (2012).

9. Tucker, C. J. Red and Photographic Infrared Linear Combinations for Monitoring Vegetation. *Remote Sens. Environ.* **8**, 127-150 (1979).

10. Huete, A. *et al.* Overview of the radiometric and biophysical performance of the MODIS vegetation indices. *Remote Sens. Environ.* **83**, 195-213 (2002).

11. Huete, A. R., Liu, H. Q., Batchily, K. & vanLeeuwen, W. A comparison of vegetation indices global set of TM images for EOS-MODIS. *Remote Sens. Environ.* **59**, 440-451 (1997).

12. Xiao, X. M. *et al.* Satellite-based modeling of gross primary production in a seasonally moist tropical evergreen forest. *Remote Sens. Environ.* **94**, 105-122 (2005).

13. Xiao, X. M., Boles, S., Liu, J. Y., Zhuang, D. F. & Liu, M. L. Characterization of forest types in Northeastern China, using multi-temporal SPOT-4 VEGETATION sensor data. *Remote Sens. Environ.* **82**, 335-348 (2002).

14. Hall, D. K., Riggs, G. A., Salomonson, V. V., DiGirolamo, N. E. & Bayr, K. J. MODIS snow-cover products. *Remote Sens. Environ.* **83**, 181-194 (2002).

15. Hall, D. K., Riggs, G. A. & Salomonson, V. V. Development of Methods for Mapping Global Snow Cover Using Moderate Resolution Imaging Spectroradiometer Data. *Remote Sens. Environ.* **54**, 127-140 (1995).

16. Xiao, X. M. *et al.* Mapping paddy rice agriculture in southern China using multi-temporal MODIS images. *Remote Sens. Environ.* **95**, 480-492 (2005).

17. Zhang, Z. *et al.* A 2010 update of National Land Use/Cover Database of China at 1:100000 scale using medium spatial resolution satellite images. *Remote Sens. Environ.* **149**, 142-154 (2014).

18. Liu, J. Y. *et al.* Spatiotemporal characteristics, patterns, and causes of land-use changes in China since the late 1980s. *J. GEOGR. SCI.* **24**, 195-210 (2014).

Figure caption


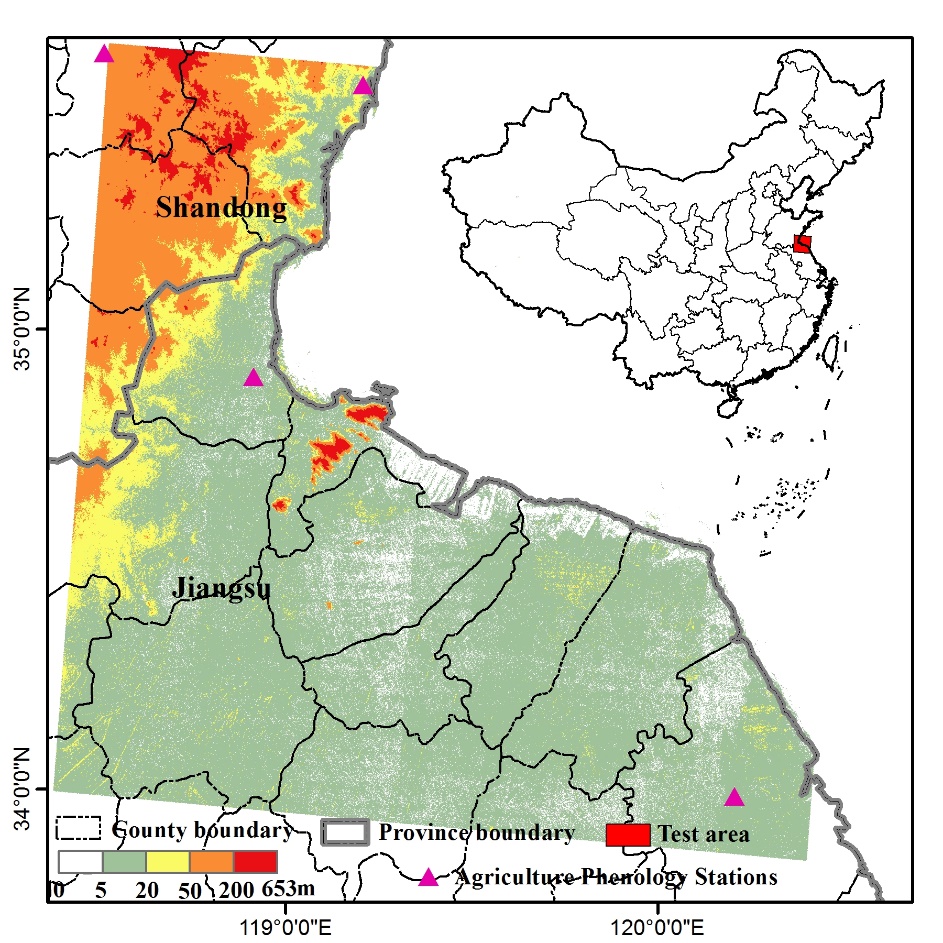


Supplementary Figure S1 Black rectangle shows the location of the test area in the Yangzi-Huaihe Plain covering the northeastern Jiangsu province and the southeastern Shandong province of China. The average elevation of this area is about 17m. Four black triangles represent the agriculture phenology stations provided by the China Meteorological Data Sharing Service System (<http://cdc.cma.gov.cn/home.do>). This map created in ArcMap 10.1.


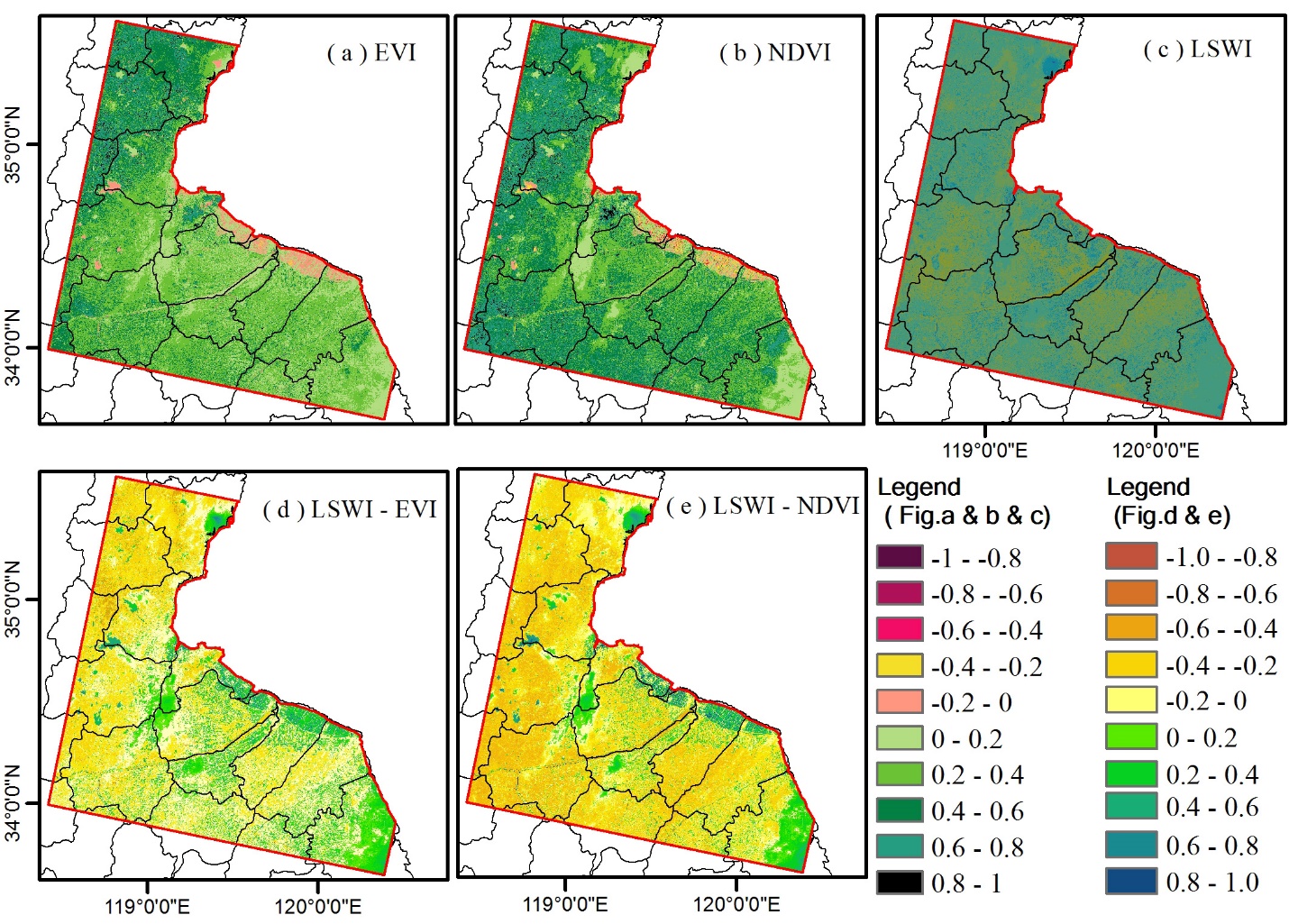


Supplementary Figure S2 Spatial characteristics of vegetation indices during the flooding/transplanting period in the test area, only including the terrestrial area. (a & b & c & d & e) show the mappings of EVI, NDVI, LSWI, LSWI-EIV, and LSWI-NVI on Julian day 191, July 10, 2013. All these maps created in ArcMap 10.1.


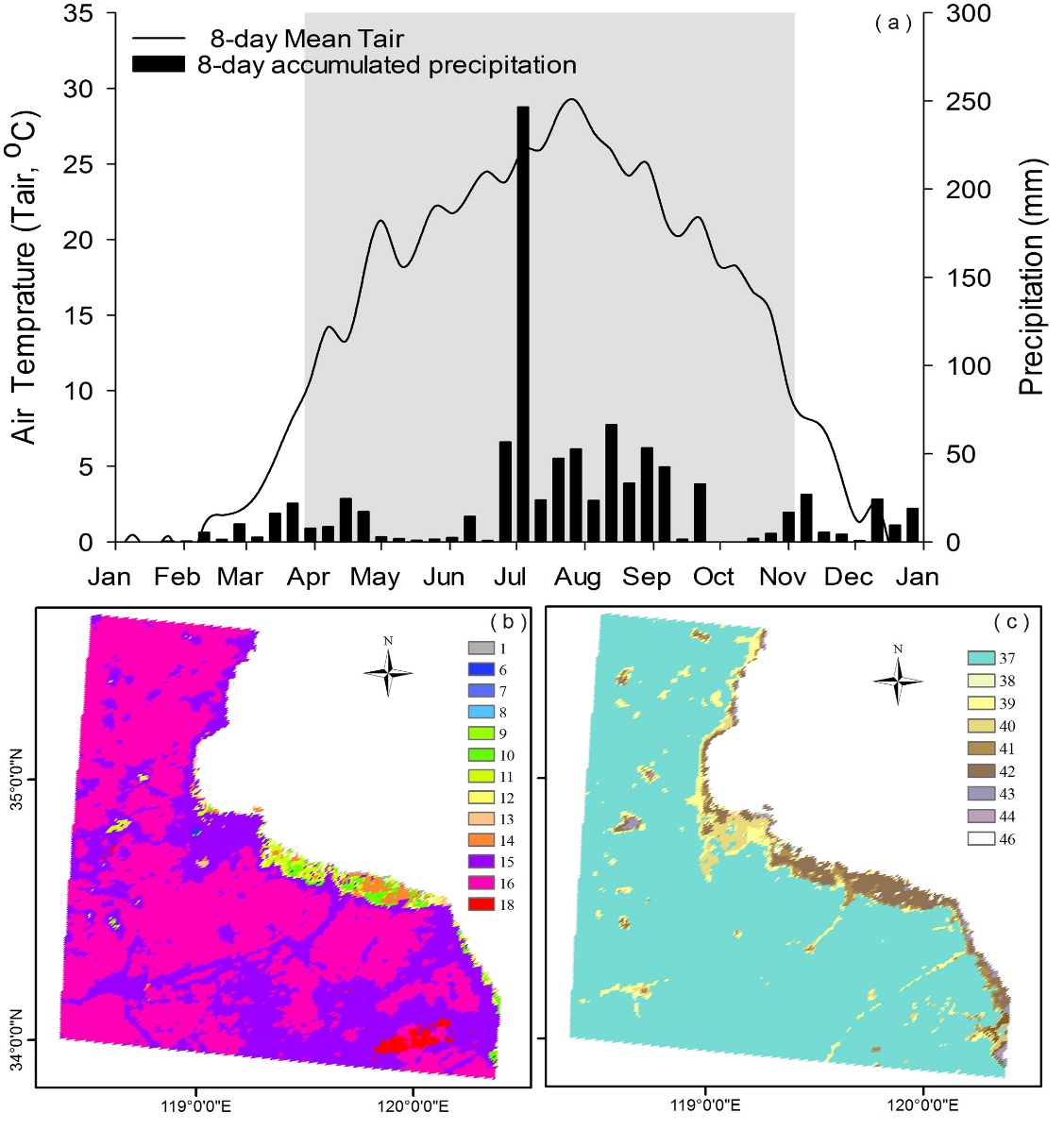


Supplementary Figure S3 (a) The seasonal dynamics of 8-day accumulation precipitation and 8-day mean air temperature, which were calculated from mean observations of four meteorological stations: Ganyu (34.833 °N, 119.117 °E), Sheyang (33.767 °N, 120.25 °E), Lvxian (35.433 °N, 119.533 °E), Rizhao (35.583 °N, 118.833 °E), with the period of T_air_ ≥10 °C highlighted. Figure S3 (b), the first date when 8-day night Land Surface Temperature (LSTnight) was greater than 5˚C. Figure S3(c) the last date when 8-day LSTnight was greater than 5˚C. They were calculated from the MYD11A2 product in 2010. Figure (a) created in SigmaPlot 12.0, Figure (b & c) created in ArcMap 10.1.


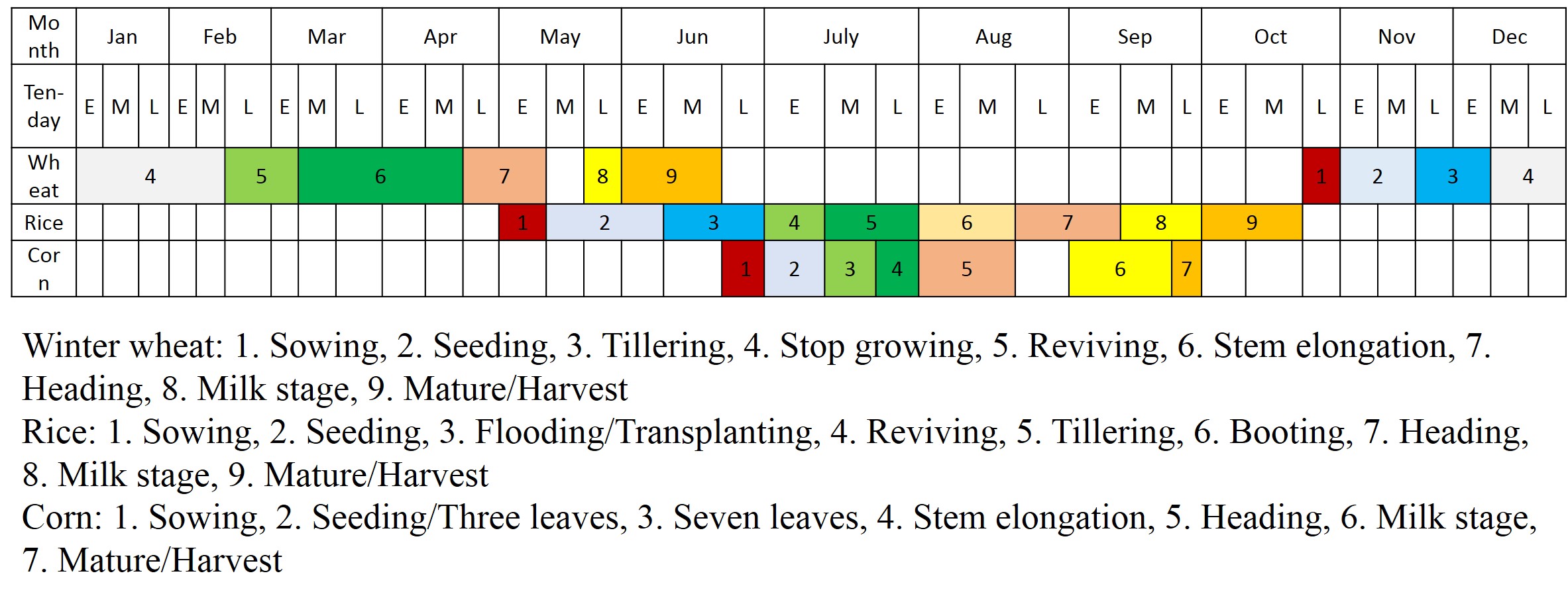


Supplementary Figure S4 Three main crop calendars include winter wheat, rice and corn, which were collected based on two agriculture phenology stations: Ganyu (34.833 °N, 119.117 °E) and Lvxian (35.433 °N, 119.533 °E). Figure created in Excel 2013.


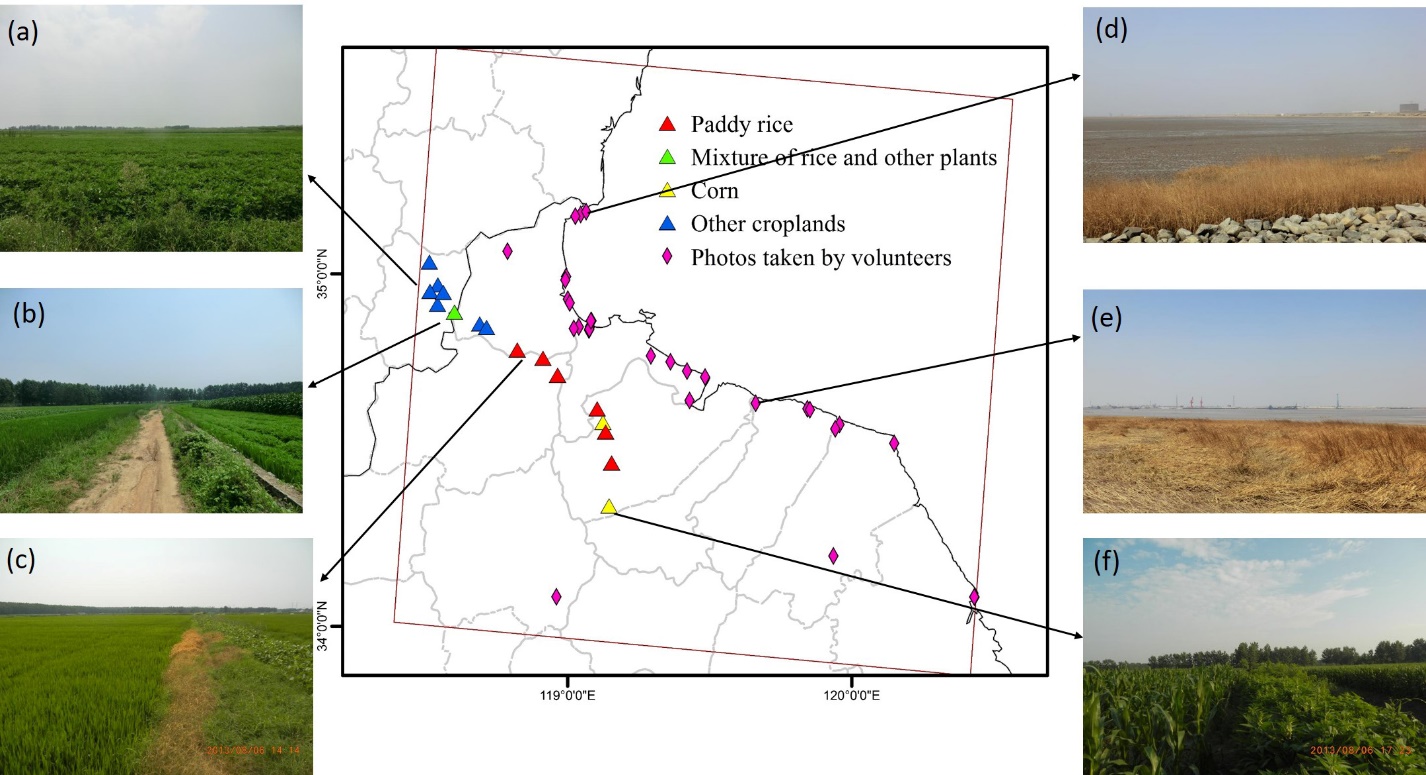


Supplementary Figure S5 Ground truth photos of different land cover types, collected by field campaigns in 2013 and volunteers of EOMF photo library (http://www.eomf.ou.edu/photos/). (a & b & c & f)- Four cropland photos taken during field campaigns in 2013 (08/06/2013) present other croplands, a mixture of rice and other plants, paddy rice fields and corn lands, respectively; (d & e)-Two photos were provided by volunteers, which were uploaded to the EOMF photo library on 06/07/2013. These two photos show the mixture of vegetation and water. Map created in ArcMap 10.1.


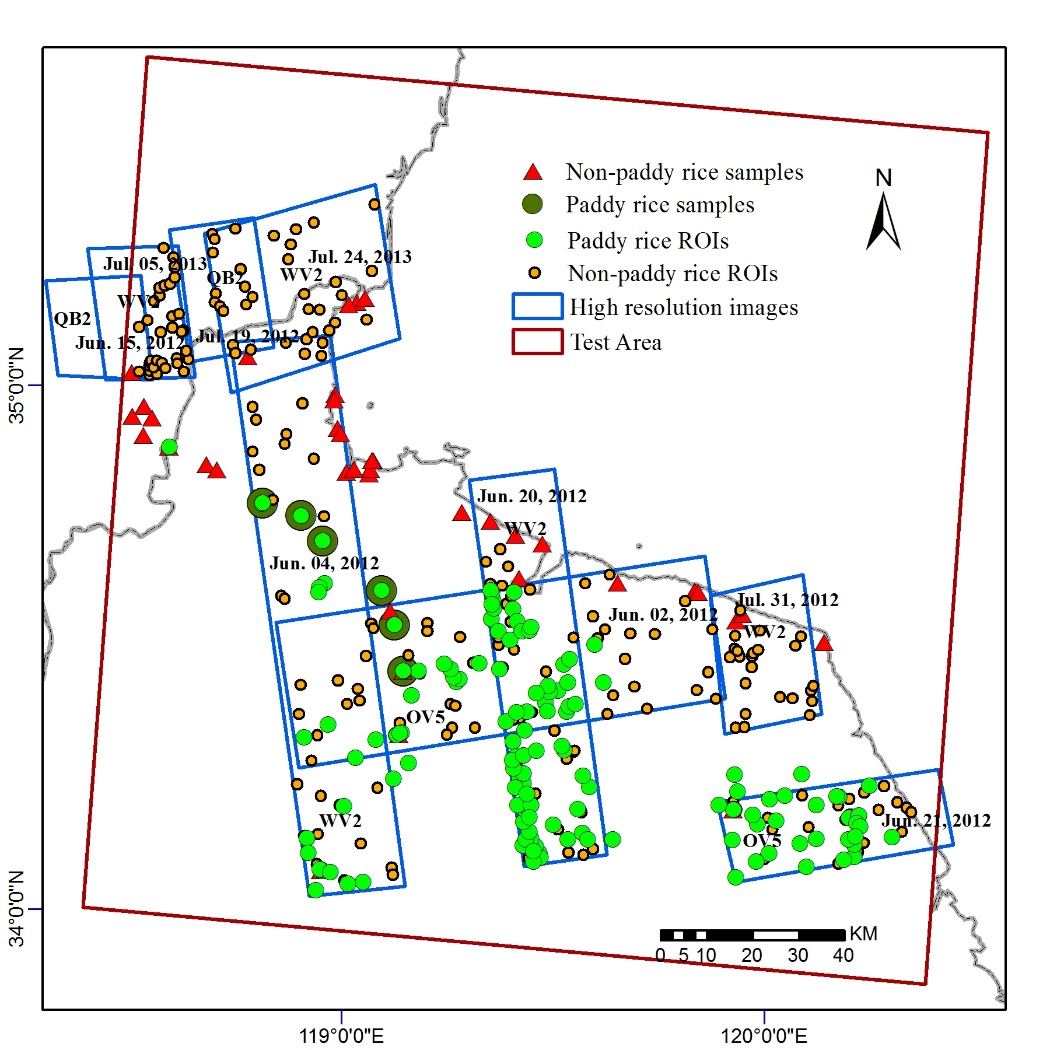


Supplementary Figure S6 Spatial distribution of ROIs used for accuracy validation of the results in this research. ROIs were drawn according to field samples, high resolution images, and Google Earth. Here, points were used to identify the location of each ROI polygon. Blue polygons show the temporal and spatial information of high resolution images used in this work. Map created in ArcMap 10.1.

Supplementary Table S1 A list of Landsat-8 images acquired in 2013 used in this study (Path/Row: 114/27). The valid percentage for each image was calculated based on cloud/cloud shadow masks generated by Fmask.

| Date | DOY | Valid percentage (%) |
| --- | --- | --- |
| April 21 | 111 | 99.2 |
| May 23 | 143 | 57.5 |
| June 08 | 159 | 35.5 |
| June 24 | 175 | 65.2 |
| July 10 | 191 | 71.9 |
| July 26 | 207 | 30.5 |
| August 11 | 223 | 88.2 |
| August 27 | 239 | 98.6 |
| September 12 | 255 | 28.7 |
| September 28 | 271 | 28.7 |
| November 15 | 319 | 98.4 |
| December 1 | 335 | 98.6 |
